# Distribution of Large and Small Dorsal Root Ganglion Neurons in Common Marmosets

**DOI:** 10.3389/fnsys.2021.801492

**Published:** 2021-12-02

**Authors:** Moeko Kudo, Sidikejiang Wupuer, Shinji Kubota, Kazuhiko Seki

**Affiliations:** Department of Neurophysiology, National Institute of Neuroscience, National Center of Neurology and Psychiatry, Kodaira, Japan

**Keywords:** dorsal root ganglion (DRG), nonhuman primate, rat, size distribution, NF200, peripherin

## Abstract

The aim of this study was to elucidate the size and distribution of dorsal root ganglion (DRG) neurons in non-human primates and to compare them with those of rodent DRG neurons. By measuring the size of NeuN-, NF200-, and peripherin-positive DRG neurons in the lumbar spinal cord of rats and marmosets, we found that the cell size distribution pattern was comparable in both species, although DRG neurons in marmosets were larger than those of rodents. This is the first demonstration that DRG neurons in marmosets have a bimodal size distribution, which has been well established in rodents and humans.

## Introduction

It is widely accepted that cell body size is an accurate marker to characterize the morphological characteristics of dorsal root ganglion (DRG) neurons (Warrington and Griffith, 1904; Lawson, 1979). Large cells are associated with thicker, myelinated Aβ sensory axons, while small cells are associated with thinner, unmyelinated C fibers (Yoshida and Matsuda, 1979; Harper and Lawson, 1985; Lawson and Waddell, 1991). Thus, a strong correlation has been observed repeatedly between DRG cell size and axonal conduction velocity (Yoshida and Matsuda, 1979; Harper and Lawson, 1985; Lawson and Waddell, 1991; McCarthy and Lawson, 1997).

Although our understanding of the cell size-dependent characteristics of DRG neurons is largely based on rodent experiments, the size of DRG cells in humans has also been measured in a few studies (Josephson et al., 2001; Feliciano et al., 2007; Zhang et al., 2017), which reported that DRG neurons are larger in humans than in rodents (Josephson et al., 2001; Haberberger et al., 2019). Anatomically, the larger size of human DRG neurons may represent a simple correlation with their larger cell bodies (Toossi et al., 2021); however, functionally speaking, larger DRG neurons are advantageous for humans to make quick sensorimotor reactions according to environmental changes using their larger bodies, if the correlation between anatomical (cell size) and functional (e.g., conduction velocity) properties is also applicable in humans. Indeed, among mammals, humans are known to possess higher tactile and kinesthetic acuity and rich behavioral repertoires compared to rodents, irrespective of body size. However, to date, the link between DRG neuron size and function (i.e., conduction speed) is less established in humans because of the difficulty in assessing their function *in vivo* (but see Pruszynski and Johansson, 2014). This limitation could be addressed by establishing a non-human primate model for analyzing DRG neuron anatomy and function. To this end, we have established electrophysiological (Umeda et al., 2012) and immunohistochemical assays for DRG neurons of non-human primates (Kudo et al., 2021). In this short report, we elucidated the size and distribution of DRG neurons in non-human primates and compared them with those of rodent DRG neurons.

## Materials and Methods

### Experimental Animals

We used 14 adult common marmosets (1–12 years old, body weight 282–409 g, four males and 10 females) and eight young male Jcl:Wistar rats (8 weeks old, body weight 251–326 g) in the present study. The animals were housed under standard conditions with food and water available *ad libitum* and a 12-h:12-h light:dark cycle. All experiments were conducted in accordance with protocols approved by the Ethics Committee for Animal Research of the National Institute of Neuroscience, National Center of Neurology and Psychiatry, Japan.

### Dissection

We anesthetized the marmosets with sodium pentobarbital (50 mg/kg) and perfused them transcardially with phosphate-buffered saline (PBS; pH 7.4), followed by 300–400 ml of 4% paraformaldehyde.

To expose the sciatic nerve, we placed the marmosets in a prone position. We cut the skin over the gluteus muscles and performed a blunt dissection to separate both heads of the biceps femoris. Once we identified the sciatic nerve below the biceps femoris, we further exposed the nerve proximally until its departure from the pelvis. Then, we removed the skin, viscera, and muscle to expose the vertebral, sacral, and medial iliac bones, which we cleared of overlying tissue in order to identify the sites of fusion of the lower lumbar vertebrae. We denoted the most caudal vertebra that lacked an articulation with a rib at its rostral margin as the first lumbar (L1) vertebra.

### Immunohistochemistry

We collected tissue from the lumbar region of the spinal cord, together with the DRG and sciatic nerve, which we post-fixed in 4% paraformaldehyde overnight at 4°C and transferred to 30% sucrose in PBS at 4°C. We cut DRG sections at 20-μm thickness on a cryostat (Microm HM550; Thermo Fisher Scientific, Waltham, MA) and mounted them on amino silane-coated slides. After we washed the sections three times with PBS, we incubated them with PBS containing 2% normal goat serum (NGS) for 1 h at room temperature, followed by incubation with a primary antibody, diluted in 2% NGS and 0.1% Triton X-100 in PBS, overnight at 4°C. Then, we washed the sections with PBS three times and incubated them with a secondary antibody, diluted in 2% NGS in PBS, for 1 h at room temperature. We washed the sections with PBS and covered them with a glass coverslip. We stained control sections using the same protocol but omitted the primary antibodies. All processes were performed in a dark chamber. We used the following primary antibodies: rabbit anti-NeuN (1:2,000; Abcam, Cambridge, MA), mouse anti-neurofilament 160/200 (NF200; 1:2,000; N2912; Sigma-Aldrich, St. Louis, MO), and rabbit anti-peripherin (1:400; AB1530; Millipore, Burlington, MA). We used the following secondary antibodies (all diluted 1:500): donkey anti-rabbit IgG (Alexa Fluor 555; Abcam) and goat anti-mouse IgG (Alexa Fluor 555; Molecular Probes, Eugene, OR).

### Histological Quantification

We acquired fluorescence images using fluorescent microscopy (BZ-X700; Keyence, Osaka, Japan) at fixed settings using a 10× or 20× objective. We performed image analysis and quantification using BZ-X710 image analysis software (BZ-H3M; Keyence). For the cell size distribution of NeuN-, NF200-, and peripherin-positive neurons, we selected every 10th DRG section spaced by 200 μm from the serial sections, and we examined four to six sections for rats or three to eight sections for marmosets in each animal. In each selected section, we measured the cross-sectional area of the labeled cells.

Before sectioning, we measured the size of lumbar DRGs (Ebraheim and Lu, 1998; Silav et al., 2016) in some of the marmosets (*n* = 4) and all of the rats (*n* = 12). We measured the L4 DRG in rats and L6 DRG in marmosets since these were the largest of those innervated by the sciatic nerve. We evaluated the cross-sectional area of the entire DRG (Beom et al., 2019) for each section of the corresponding DRG.

### Statistical Analysis

We tested the difference of DRG cell size for each neural marker (NeuN, NF200, and peripherin) between rats and marmosets by either the Wilcoxon rank-sum test (for mean cell size) or a two-sample Kolmogorov–Smirnov test (for the distribution pattern). We considered *p* < 0.05 as significant in all statistical analyses.

## Results

**Figure 1:**
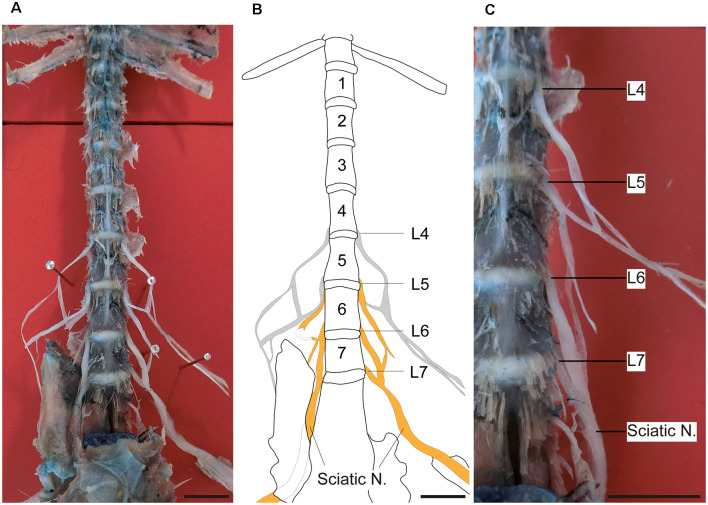
Sciatic nerve and DRGs of marmosets. Ventral views of the dissection and reference line drawing made from a marmoset, showing the sciatic nerve and its segmental origins. **(A)** Photograph showing the lower thoracic and lumbar spinal cord. The sciatic nerve is formed by the combination of several nerves in the lumbar spine. **(B)** A line drawing of the photograph shown in **(A)**. The lumbar region of this specimen contained seven vertebrae. The L5, L6, and L7 spinal nerves contributed to the sciatic nerve. Gray lines: nerves hidden by the overlying pelvic bone. **(C)** Right side of the lower lumbar vertebrae as shown in **(A)** (enlarged). Orange: sciatic nerve. Scale bars: 10 mm.

Figure 1 shows the sciatic nerve and its segmental origin in marmosets. In this dissection, the segmental origin of the sciatic nerve, together with those of the femoral and saphenous nerves, were clearly visible (Figure 1A). We found that the sciatic nerve of marmosets originated mainly from the 5th, 6th, and 7th lumbar spinal roots (Figures 1B,C). This result was different from that observed in rodents, which originates from the 3rd and 4th (mice) or 4th and 5th (rats) lumbar spinal roots (Rigaud et al., 2008). Therefore, in our comparison of DRG neuron size, we focused on the DRGs of L4–6 in rats and L5–7 in marmosets.

**Figure 2:**
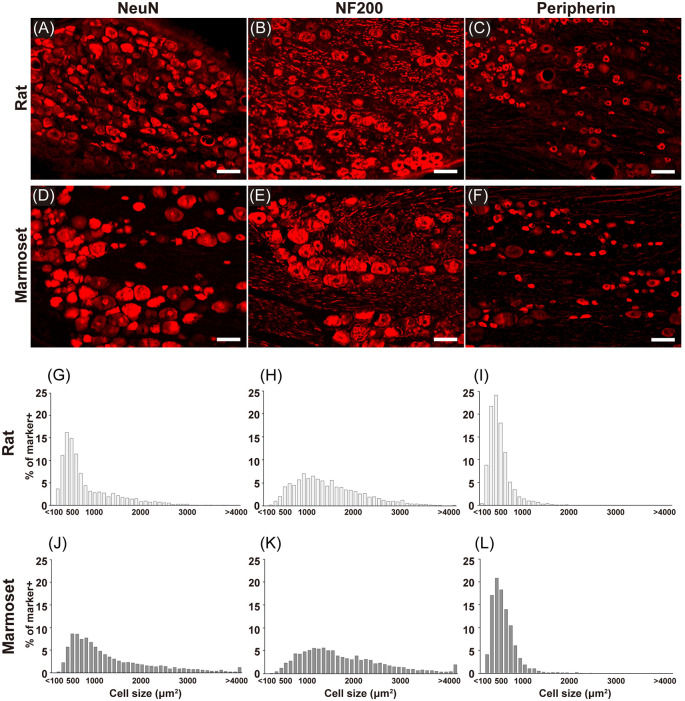
Immunohistochemistry. Immunostaining for NeuN **(A,D)**, NF200 **(B,E)**, and peripherin **(C,F)** in the DRGs of rats **(A–C)** and marmosets **(D–F)**. Histograms showing the size distribution of NeuN-** (G,J)**, NF200- **(H,K)**, and peripherin-positive **(I,L)** cells. Scale bars: 100 μm.

Figure 2 shows examples of immunostained sections containing DRG neurons in rats (Figures 2A–C) and marmosets (Figures 2D–F). We stained each DRG slice for NeuN (Figures 2A,D), a neuronal marker, NF200 (Figures 2B,E), a marker for myelinated primary afferents that convey somatosensory signals other than nociception (Ma, 2002), and peripherin (Figures 2C,F), a marker for unmyelinated primary afferents that convey nociceptive signals (Amaya et al., 2000). In these examples, cells with a larger diameter were found frequently in sections of marmoset DRGs (Figures 2D–F). Then, we evaluated the size of each DRG cell stained in each section from all animals (shown in Figures 2G–L). In general, we confirmed the well-established bimodal distribution of DRG cell body size (Warrington and Griffith, 1904; Lawson, 1979; Ohnishi and Ogawa, 1986; Schmalbruch, 1987; Verge et al., 1990) in rats (Figures 2G–I). In rats, the size distribution of DRG cells labeled with NF200 (Figure 2H) was comparable with that of large “light” cells, which were also labeled with RT97, another antibody specific for this subpopulation of DRG neurons (Lawson et al., 1984). The peripherin-labeled DRG neurons tended to be small (Figure 2I), which again is comparable with the distribution of small “dark” cells. These large and small DRG subpopulations seemed to underlie the bimodal property of NeuN-labeled DRG neurons (Figure 2G). The cell type distribution of marmosets (Figures 2J–L) was comparable with that of rats. The cell size distribution patterns of the peripherin-positive and NF200-positive neurons were not different between rats and marmosets (two-sample Kolmogorov–Smirnov test, *p* = 0.996 and *p* = 0.101, respectively, Figure 2), which fundamentally composed the bimodal distribution of NeuN-positive neurons. This result suggests that the bimodal cell size distribution of DRG neurons reported in rodents is also applicable to marmoset DRG neurons.

**Figure 3:**
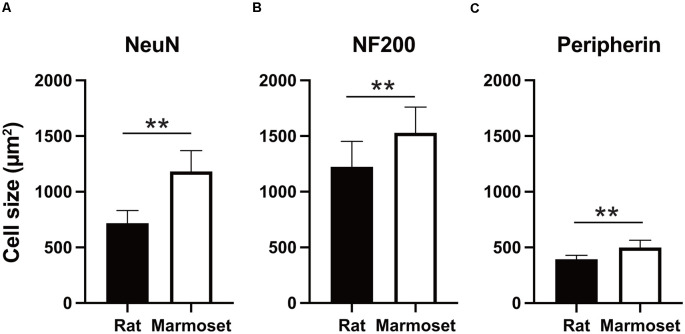
Cell size comparison. Comparison of the size of NeuN- **(A)**, NF200- **(B)**, and peripherin-positive cells **(C)** between rats and marmosets. **p < 0.01.

Figure 3 summarizes the average size in each subcategory of DRG cells in rats (black) and marmosets (white). The average size of NeuN-positive DRG neurons in rats was 716.9 ± 114 μm^2^ (*n* = 3,902 cells), which was significantly smaller than that in marmosets (1,181.7 ± 188.3 μm^2^, *n* = 6,422 cells, *p* < 0.005; Figure 3A). Similarly, we found significant differences in the average size of NF200- or peripherin-labeled cells between rats and marmosets. The average size of NF200-positive DRG neurons in rats and marmosets was 1,224.1 ± 288.8 μm^2^ (*n* = 2,290 cells) vs. 1,528.6 ± 231.8 μm^2^ (*n* = 3,677 cells), respectively (Figure 3B), and the average size of peripherin-positive DRG neurons was 393.6 ± 34.8 μm^2^ (*n* = 2,658 cells) vs. 499.8 ± 64.4 μm^2^ (*n* = 3,295 cells), respectively (Figure 3C). Therefore, we concluded that DRG cell size was generally larger in marmosets, but it may not be ascribed to the biased distribution of neither large nor small DRG cells.

**Figure 4:**
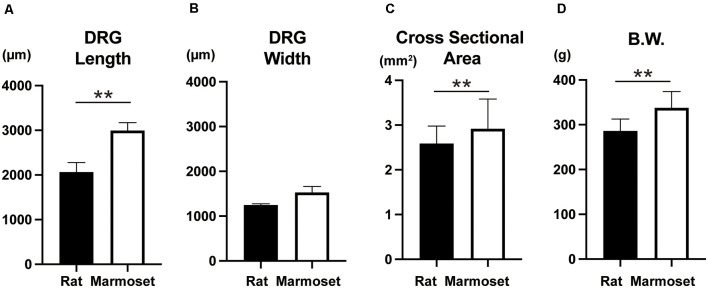
Whole DRG size and body weight. Comparison of the whole DRG length **(A)**, width **(B)**, cross-sectional area **(C)**, and body weight **(D)** between rats (*n* = 8) and marmosets (*n* = 4 for **A**,**B**,**C** and *n* = 12 for **D**). **p < 0.01.

The size of DRG neurons in rats and marmosets are shown in Figures 4A,B,C. The results showed that marmosets possessed larger DRG neurons, which were especially longer than those in rats. Since body weight (Figure 4D) was also larger in marmosets, the size of DRG neurons might increase as a function of body size.

## Discussion

In this study, we found that the sciatic nerve of marmosets originated mainly from DRGs of the 5th, 6th, and 7th lumbar spinal cord. Compared with the DRGs of rodents, in which the sciatic nerve originates from the 3rd and 4th (mice) or 4th and 5th (rats) lumbar spinal roots (Rigaud et al., 2008), we suggest that the segmental origin of the sciatic nerve in marmosets is shifted caudally. A number of studies have compared the anatomy of peripheral nerves for the lower limbs between macaque monkeys and rodents (Janjua and Leong, 1984, 1987; Yeong et al., 1998). These studies consistently reported that the segmental origins of the sciatic nerve or its downstream nerves are biased caudally in macaques compared to rats. For example, sciatic neurons are distributed to L4–7 in macaques and L3–6 in rats (Janjua and Leong, 1984). Interestingly, the human sciatic nerve originates from more caudal segments (L5–S1; Baumer et al., 2015). These comparisons among rodents, marmosets, and humans suggest that the caudal shift of sciatic nerve innervation correlates with the evolutionary status of vertebrates. Since the segmental origin of the sciatic nerve in marmosets is similar to that of macaques, which is closer to the characteristics of humans compared to rodents. The common marmoset (*Callithrix jacchus*) has attracted considerable attention in the research fields of biomedical science (Okano et al., 2012) and behavioral science (Prins et al., 2017). Furthermore, transgenic marmoset models of human disease have been developed recently (Sasaki et al., 2009; Sato et al., 2016; Tomioka et al., 2017). Our results add another example to the similarities between marmosets and humans and suggest that marmosets could be an animal model for human peripheral nerve disorders and their treatment (Kudo et al., 2021).

In this study, we confirmed the bimodal size distribution of large and small DRG neurons in marmosets. Since comparable observations have been reported repeatedly in other species, this result was expected. Nevertheless, this finding provides invaluable information for future studies using New World monkeys as a model of the human peripheral nervous system and for the establishment of therapeutic strategies for neuronal diseases such as chronic pain syndrome. In contrast, we found that marmoset DRG cells were larger than those of rats (Figure 3), which is in agreement with previous studies comparing humans and rodents (Josephson et al., 2001; Haberberger et al., 2019). We hypothesize that this finding may provide support for the higher acuity and cognitive function of non-human primates for non-noxious inputs from the lower limbs compared to rats, because the relative increase in the size of large DRG neurons, and thus myelinated afferents, will increase conduction velocity in non-human primates. However, we found no difference in the size of two subpopulations of DRG neurons between rats and marmosets. This result may suggest that the size of DRG neurons with myelinated and nonmyelinated axons is increased proportionally, and thus affects the cognitive profile to noxious and non-noxious sensory inputs comparably. We rather suggest that the difference in cell size between rats and marmosets may simply correspond to the greater size of the body and peripheral nervous system of marmosets, which leads to the requirement for sensory nerves with higher conduction velocities to perform proper sensorimotor actions using their larger bodies and limbs.

## Data Availability Statement

The original contributions presented in the study are included in the article, further inquiries can be directed to the corresponding author.

## Ethics Statement

The animal study was reviewed and approved by the Ethics Committee for Animal Research of the National Institute of Neuroscience, National Center of Neurology and Psychiatry, Japan.

## Author Contributions

MK and SW performed the dissections. SW and MK performed the histological analyses. SK and MK performed the statistical analyses. KS conceived and designed the study and was responsible for the experiments, analyses, and interpretation of the data, as well as manuscript drafting, editing, and revising.

## Conflict of Interest

The authors declare that the research was conducted in the absence of any commercial or financial relationships that could be construed as a potential conflict of interest.

## Publisher’s Note

All claims expressed in this article are solely those of the authors and do not necessarily represent those of their affiliated organizations, or those of the publisher, the editors and the reviewers. Any product that may be evaluated in this article, or claim that may be made by its manufacturer, is not guaranteed or endorsed by the publisher.
